# Clinal variation as a tool to understand climate change

**DOI:** 10.3389/fphys.2022.880728

**Published:** 2022-10-11

**Authors:** Harshad Vijay Mayekar, Durga Kavya Ramkumar, Divita Garg, Abhishek Nair, Ashwin Khandelwal, Kavya Joshi, Subhash Rajpurohit

**Affiliations:** Division of Biological and Life Sciences, School of Arts and Sciences, Ahmedabad University, Ahmedabad, GJ, India

**Keywords:** Clines, latitudes, altitudes, climate change, temperature, clinal shift, rapid adaptation, *Drosophila melanogaster*

## Abstract

Clines are observable gradients that reflect continuous change in biological traits of species across geographical ranges. Clinal gradients could vary at geographic scales (latitude and altitude). Since clinal variations represent active genomic responses at the population level they (clines) provide an immense power to address questions related to climatic change. With the fast pace of climate change i.e. warming, populations are also likely to exhibit rapid responses; at both the phenotypic and genotypic levels. We seek to understand how clinal variation could be used to anticipate climatic responses using *Drosophila*, a pervasively used inter-disciplinary model system owing to its molecular repertoire. The genomic information coupled with the phenotypic variation greatly facilitates our understanding of the *Drosophilidae* response to climate change. We discuss traits associated with clinal variation at the phenotypic level as well as their underlying genetic regulators. Given prevailing climatic conditions and future projections for climate change, clines could emerge as monitoring tools to track the cross-talk between climatic variables and organisms.

## 1 Background

**FIGURE 1:**
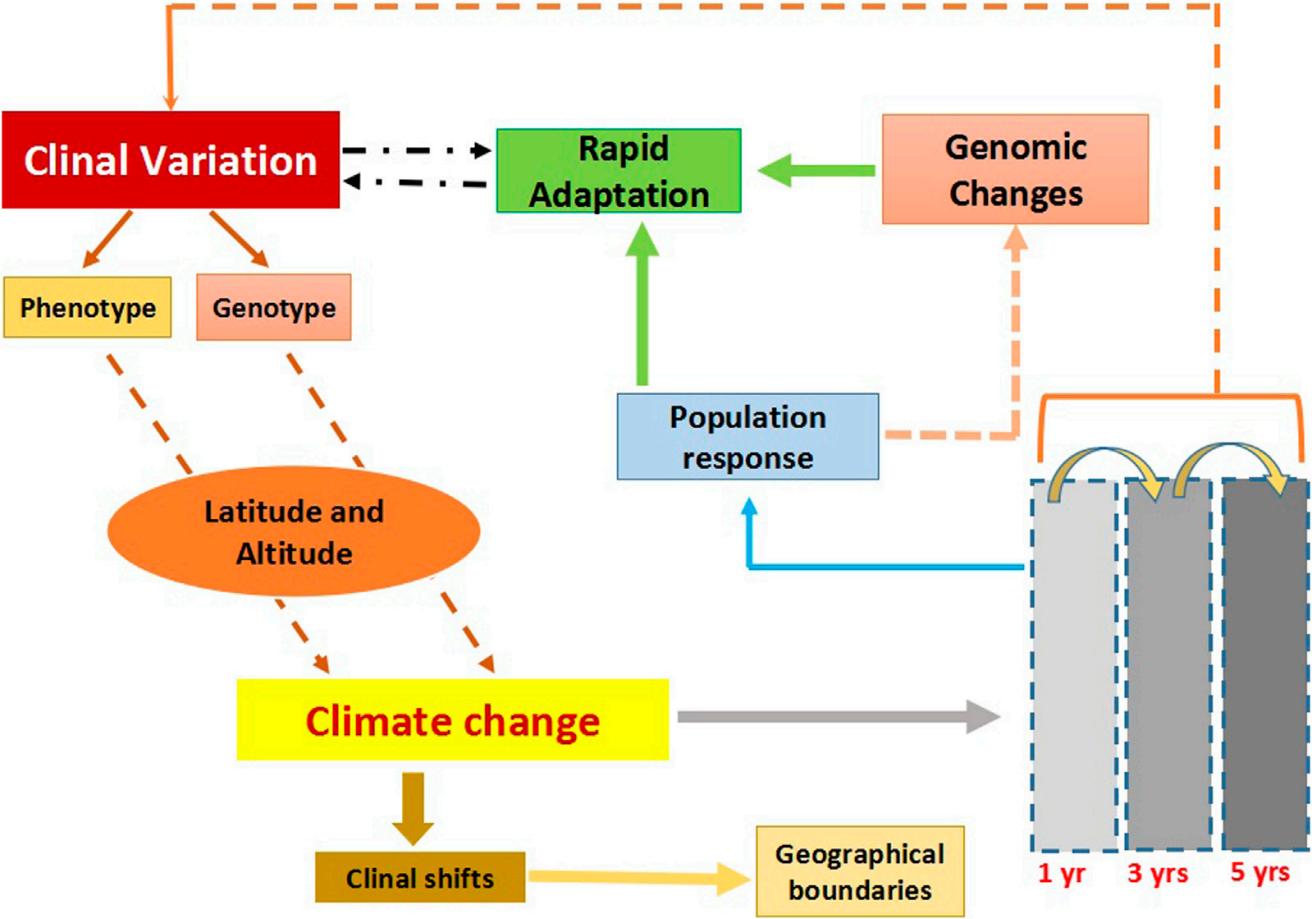
The flowchart describes clinal variation and the impact of climate change on clines. Clinal variation can occur both at the phenotypic and genotypic level across different geographic scales; altitude and latitude. Climate changes are known to influence existing clines, resulting in clinal shifts in which species either expand or contract in their habitat range. Response to climate change could be rapid and these could be tracked within a span of few years. Populations responding to climate change at the genomic level could modify existing ones. Overall clines could be a tool to track rapid environmental changes.

Species extinctions have been common in the history of this planet (Bambach 2006; Qiao et al., 2016). Striking among these extinctions has been the global loss of megafauna (weighing more than 45 kg) which began approximately fifty thousand years ago, towards the end of Pleistocene and continued into the Holocene (Koch and Barnosky 2006). Termed as the Late Quaternary Extinction (LQE), this time period happened to coincide with the latest glacial-interglacial transition, raising considerable debate about the anthropogenic versus climatic impacts on species extinctions (Barnosky and Lindsey 2010; Nogués-Bravo et al., 2010; Sandom et al., 2014). Even then growing consensus indicates a synergistic role of both climatic and anthropogenic influences on species extinctions (Prescott et al., 2012). Anthropogenic influence could likely be postulated through overhunting (Flannery 1990), fire usage (Miller et al., 2005) and introduction of predators (Alroy 2001) with the onset of human colonization across continents. The climate-centric perspective hypothesized habitat loss, seasonal alterations affecting nutritional availability from plants to be some of the causes behind species extinctions [reviewed in (Koch and Barnosky 2006)]. Temperature has been a key driver implied in climatic changes; specifically decreasing temperatures during the last glacial maxima have been associated with megafaunal extinctions (Stewart et al., 2021). The fast pace of glacial-interglacial transition, the large size (≥45 kg) of the mega-fauna added could have limited the time to adapt or migrate (Koch and Barnosky 2006). Arguably, recent climate change confined to the limited time frame from 1955 to 2022 has been more appalling, due to its enormous extinction rate (Akçakaya et al., 2014). Climate change consequences (Cahill et al., 2013) can be seen as an overall reduction in biodiversity (Bellard et al., 2012; Meyer et al., 2022), as well as spatial redistributions of organisms globally (Sunday et al. 2012). While species extinction is a reality, rapid climate change almost always does not imply the doom for species. Organismal response to rapid climate change varies and is predominantly evident through modifications of life history traits (Hoffmann and Sgrò 2011), shift in reproductive timings and migration (Scheffers and Gretta 2019; Parmesan and Singer 2022). Mammalian extinction could be striking because of its (mammals’) sheer size, yet ectotherms (sensitive to temperature fluctuations) could be immediate targets of climate change. The speciose insects could particularly provide valuable insights regarding extinction events and climate vulnerability. Although, fossil records, carbon dating and molecular phylogeny address extinction events partially, inferences can be equivocal. We propose that clines can be used to decipher/predict the response of organisms to changing climate (Figure 1).

The term “cline” was introduced by Julian Huxley (1938) and is generally defined as an observable gradient in a biological characteristic across a larger geographical range. A variety of species exhibit geographic variation, i.e., systemic changes in form, size or any other characteristic along the environmental gradient (Koch 1986). For this review we broadly discuss *Drosophila* genus and their relative potential to understand adaptive response to climate change.

**FIGURE 2:**
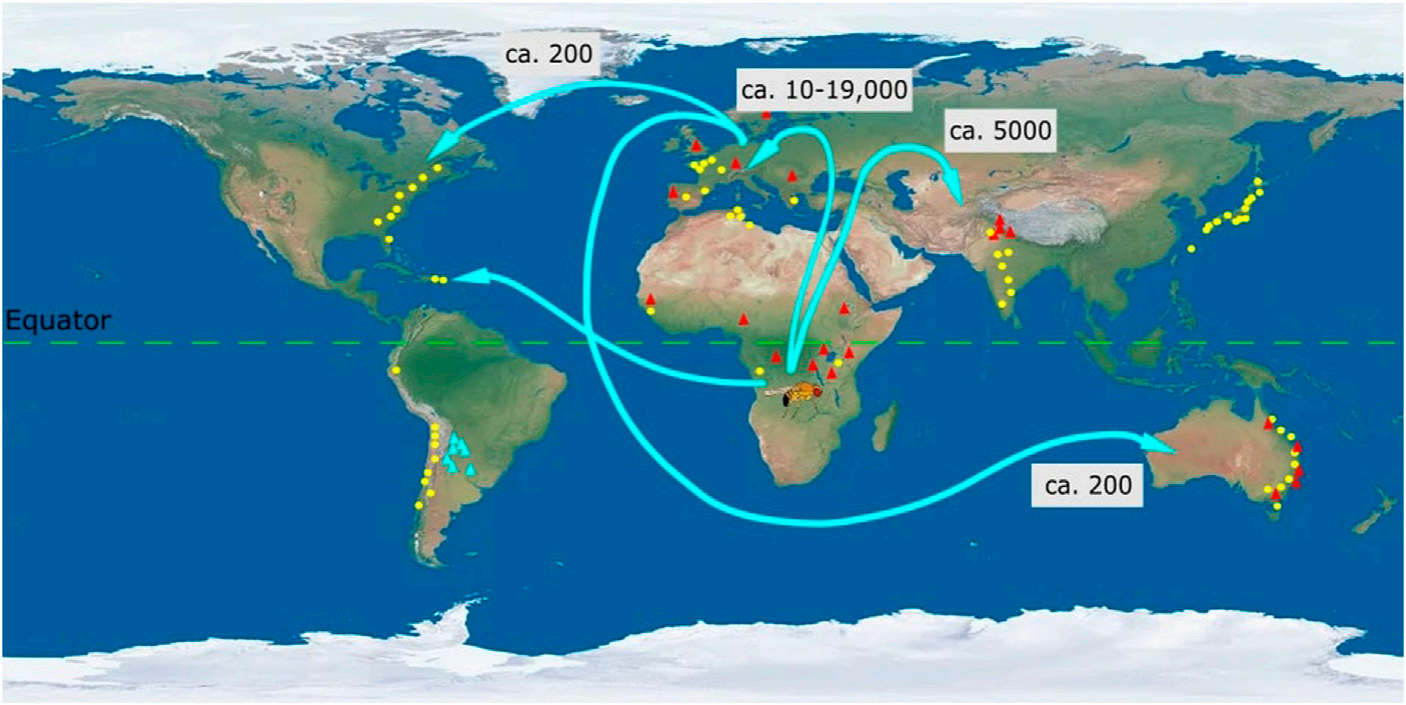
Map of the direction of spread and clinal variation of *Drosophila melanogaster*. Ancestral populations of *Drosophila melanogaster* from sub-Saharan Africa (Zambia) dispersed to Europe (ca. 10-19,000 years ago) and Asia (ca. 5,000 years ago) (David and Capy 1988; Haudry et al., 2020). From Europe there were subsequent dispersals to Australia and North America [<200 years ago (Keller, 2007)]. Population recently dispersed from Africa to North America and this dispersal is considered to be mediated through human movement. Clinal variation in *D. melanogaster* across latitudes and altitudes has been denoted by yellow circles and red triangles, respectively. Each circle or triangle represents particular geographic locations (not to scale) from which populations were collected. Latitudinal and altitudinal clines have been observed for *D. melanogaster* in Africa, Europe, Asia, and Australia. In North and South America only latitudinal clines are known for *D. melanogaster* and no clines are known across altitudes. Instead *D. buzzati* (blue) exhibits an altitudinal cline (thermal tolerance) in South America (Sørensen et al., 2005). Numbers in boxes represent the approximate time in years when *D. melanogaster* dispersed throughout the world from the ancestral population in Africa. Latitudinal clines have been depicted for desiccation tolerance (Asia: India (Karan et al., 1998)), chromosomal inversion (Asia: Japan (Inoue and Watanabe 1979)) and wing area (Australia, (James et al., 1995)), (South America, (Land et al., 1999)). Altitudinal clines have been depicted for body pigmentation (Asia (Parkash and Munjal, 1999)), diapause incidence (North America (Schmidt and Conde 2006)) and cold tolerance (Australia, (Collinge et al., 2006)). Latitudinal and altitudinal clines for wing area mirror each other in Europe and Africa (Klepsatel et al., 2014). All traits depicted exhibit positive clinal variation. Thus, wing area is larger for populations at higher latitudes and altitudes compared to populations at lower latitudes and altitudes (Klepsatel et al., 2014).

*Drosophila melanogaster* (commonly known as vinegar fly) originated in Central and South Africa from where it dispersed to Europe, Asia and more recently to North America, and Australia (David and Capy 1988; Haudry et al., 2020) (see Figure 2). Within a span of 20,000 years *D. melanogaster* as with other species of the genus adapted to local climate regimes. This can be seen through the wide range of clines across species spread across continents (Table 1 and Supplementary Table S1). We discuss clines broadly categorized under morphology; reproduction, development and behaviour; stress resistance, and metabolic traits.

**TABLE 1:** Latitudinal and altitudinal clines in *Drosophila* species have been presented from six continents. Each trait exhibiting a cline is represented by a coded colour symbol (refer to box below). Clines are broadly categorized as: morphological, reproduction, development and behaviour, stress resistance and energy reserves. There is considerable parallelism as well as variation for the same trait across continents. Traits for clines are codes with different symbols and colours.

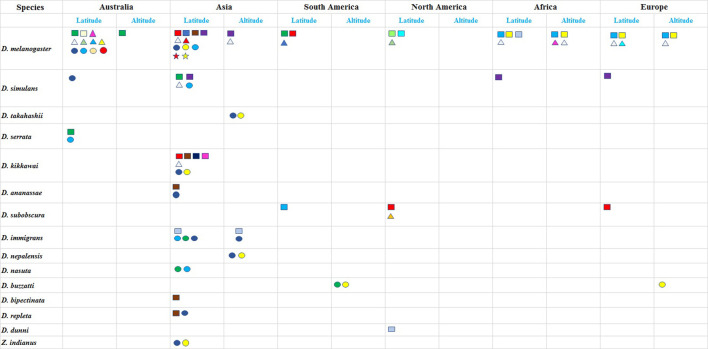				

Body size, 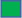; Wing length, 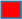; Wing loading, 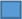; Cell number, 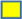;Trident pigmentation, 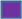; Body weight, 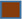; Thorax length, 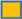; Abdominal bristles, 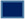; Sterno-pleural bristles, 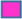; Aldh-Phe gene, 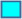; In(3R) P, 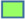; Wing size, 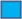; Abdominal pigmentation, 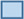; Wing-thorax ratio, 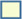; Ovariole number, 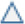; Copulation period, 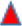; Egg size, 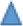; Diapause, 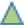; Thermoregulation, 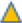; Larval developmental time, 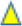; Circadian rhythm, 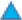; Time less allele, 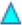; Mid day siesta, 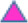; Desiccation resistance, 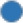; Starvation resistance, 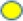; Heat resistance, 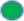; Wolbachia infection frequency, 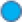; Trehalose, 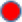; Chill recovery, 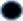; Cold resistance, 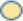; Ethanol tolerance, 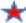; Total body lipids, 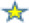

## 2 Clinal variation

### 2.1 Clines for morphological and reproductive traits

#### 2.1.1 Latitudinal clines

Clinal variations are influenced by environmental variables which co-vary with latitude and have been observed in a variety of ectotherms/stenotherms, which comprise various species of the genus *Drosophila.* As per Bergmann’s rule, body size is large in colder habitats (lower temperatures), and so does body form and shape (e.g., appendices) (Allen’s rule). These rules were founded on the basis of endotherms (Köhler et al., 2017) but also find reflection in a number of altitudinal/ latitudinal clines for ectotherms. (Ray 1960; Atkinson 1994). Alluding to Bergmann’s rule, latitudinal clines in *D. melanogaster*, are known for several traits, viz., body size (David and Charles 1975), larval growth rate (Robinson and Partridge 2001), developmental rate (James et al., 1995), ethanol tolerance, ovariole number (David and Charles 1975), abdominal pigmentation (Rajpurohit et al., 2008a), cold tolerance, heat and starvation tolerance (Da Lage et al., 1990) (refer Table 1). While traits of body size, ovariole number and cold tolerance exhibit positive clinal variation, i.e., increase with increasing latitude, the trend reverses for larval development or heat tolerance.

*D. melanogaster* populations obtained from the coasts of Australia and South America exhibited clines for egg size (Klepsatel et al., 2014) with egg size increasing with latitude. When all populations were maintained at constant temperatures, an increase in egg size was recorded with corresponding increase in latitude. Parallel differentiation (Adrion et al., 2015) wherein clinal trends are similar across geographical scales (also see Figure 2) suggests a possible overlap of proximate influential factors [e.g., temperature across latitudes (Klepsatel et al., 2014) (Figure 2)].

The magnitude of clinal variation also differs for latitudinally similar gradients. Photoperiod and temperature influence circadian rhythms and diapause especially in temperate species. Shorter photoperiods and lower temperatures induce diapause, i.e., winter dormancy (Danilevsky et al., 1970; Schmidt and Conde 2006). Even then, North American *D. melanogaster* populations, show an increase in diapause frequency compared to East Australian populations with respect to increasing latitudes (Collinge et al., 2006).

Pigmentation, a highly variable trait across species, also exhibits latitudinal clinal variation in the *Drosophila* genus. Trident pigment pattern exhibited a positive cline with respect to latitude in both *D. melanogaster* (David et al., 1985) and *D. simulans* (David and Capy, 1988; Capy et al., 1988). However, the slope was steeper in the case of *D. melanogaster* compared to *D. simulans* with extremely low temperatures inducing trident pigmentation. Another trait influenced by latitude is female remating or polyandry (Taylor et al., 2016). A study investigating the effect of temperature on mating in female *D. pseudoobscura* populations indicated that polyandry follows a latitudinal cline. It was found that females re-mated at an increased rate in a colder environment corresponding to a higher rate of polyandry at higher latitudes. However, genetic factors also had an impact on the latitudinal cline in this species (Taylor et al., 2016). Latitudinal clines partly result from spatially varying selection, in many cases temperature being considered the major selective factor (Stalker and Carson 1947). In an extensive study across latitudes in the Indian subcontinent (Rajpurohit and Nedved 2013) body size, ovariole number, starvation tolerance, and several other traits. were found to vary with latitude. Trait variation in this case is largely speculated to be driven by temperature.

#### 2.1.2 Altitudinal clines

Altitudinal clines are assumed to mimic latitudinal clines qualitatively since mean temperature reduces as a result of increasing altitude and latitude (Figure 2). Altitudinal clines also exist for many morphological traits across a variety of species. Body color/size can also change along altitudinal gradients. The “thermal melanism” hypothesis states that organisms in colder regions, i.e., higher latitudes or higher altitudes are usually darker compared to the ones in the warmer regions for efficient thermoregulation (Trullas et al., 2007). In *D. melanogaster* populations from different continents, thoracic pigmentation correlates with latitudes. Flies from high altitudes have darker body pigmentation thus correlating with latitudinal clines (Pitchers et al., 2013). Positive altitudinal cline for body size have been identified in *D. buzzatti* (Dahlgaard et al., 2001), and *D. robusta* (Stalker and Carson 1948).

A similar cline has also been recorded in *D. melanogaster* populations collected from highland and lowland regions in the Indian subcontinent (Parkash et al., 2008). *D. melanogaster* populations at higher altitudes were more melanized than populations from lower latitudes. Darker flies with more than 45% melanization were found to be more resistant to desiccation than lighter flies with less than 30% melanization. The phenotypic variations exhibited by these organisms were concluded to correspond with geographical factors such as latitude and altitude as well as changing weather conditions like annual temperatures and humidity (Parkash et al., 2008). Other *Drosophila* species known to have similar reported clines for pigmentation include *D. simulans* (David and Capy 1988) and *D. dunni* (Hollocher et al., 2000) (Supplementary Table S1).

Clinal patterns across latitudes do not always reflect those of altitudes. *D. melanogaster* females collected from highlands (3,000 m) in Ethiopia (Africa) exhibited bigger thorax sizes than females collected from lowlands (525 m) (Klepsatel et al., 2014). At higher altitudes, selection in addition to temperature, e.g., lowered oxygen availability could also affect body size (Frazier et al., 2001). That is lowered oxygen levels (hypoxia) could induce smaller body size. In that case, larger thorax in the highland Ethiopian population presents an example of counter-gradient variation (Conover and Schultz 1995) wherein genotypes give rise to phenotypes in contrast to environmental variation. Thus even under hypoxic conditions at high altitude, the joint effect of temperature was correlated with larger body size.

### 2.2 Stress resistance and energy reserve clines

High altitudinal species *D. takahashii* and *D*. *nepalensis* were more desiccation tolerant than their low altitude counterparts (Parkash et al., 2005). Alternately, low altitudinal populations were more starvation tolerant than the high altitudinal ones. *D. nepalensis,* which is more colder adapted than *D. takahashii* exhibited higher tolerance to desiccation/starvation stressors than *D. takahashii.* The link between higher altitudes and physiological traits (e.g., body size, pigmentation, stress response) need further investigation.

The maintenance of body processes requires a threshold amount of energy which is reflected as the standard metabolic rate (Norin and Metcalfe 2019). The metabolic rate of an organism indicates its ability to grow and reproduce, which relates to overall performance, making it a possible target for selection. There are several hypotheses in existence that explore the relationship between the metabolic rate and the longevity of an organism. For example, the “compensation hypothesis” (Pettersen et al., 2018) states that organisms with a lower standard or basal metabolic rate are at an advantage since it results in lower maintenance costs, which allows allocation of energy towards functions such as reproduction. In contrast, higher standard or basal metabolic rates allow the sustenance of larger organs, which may contribute to higher reproductive yield. This is known as the “increased-intake hypothesis.” The variance in metabolic rate has been found to be heritable, and hence expected to evolve (Norin and Metcalfe 2019).

Energy acquisition by ectotherms is restricted by their ability to commute to acquire resources. Plasticity in metabolism can only be considered advantageous if there is a balance between energy acquisition and energy expenditure which can be modulated as per the energy demand. Metabolic plasticity could prove beneficial since it takes environmental cues into consideration and adjusts life-history accordingly; less resources means lesser energy expenditure, abundant resources means enhanced growth (Auer et al., 2015). This makes metabolic plasticity a likely clinal candidate to investigate in the face of climate change (Norin and Metcalfe 2019).

The “metabolic cold adaptation hypothesis” posits that in a set thermal range, ectotherms originating from chilly climates will have a higher metabolism than those from hotter environments (Carrasco and Antonio, 2004). This hypothesis also assumes that populations from higher latitudes are more sensitive to changes in ambient temperature and would therefore be more likely to rapidly cope with rising temperatures (Nielsen et al., 1999). Studies have shown that flies selected for rapid recovery from chill comma have increased metabolic rates and are able to suppress their metabolic rates and maintain homeostasis in response to chronic cold exposure. Indeed numerous studies have suggested that latitudinal clines for allele frequency of central metabolism-related enzymes are products of selection brought by climate change or seasonal fluctuation (Williams et al., 2014). On the Indian subcontinent *Drosophilidae* exhibit differences in storage as well as utilization of energy metabolites carbohydrate and lipids. However, storage and utilization of metabolites may differ under different stressful conditions such as desiccation and starvation. In order to cope with physiological stresses, adaptive responses in insects involve metabolite storage, e.g., trehalose (Chippindale et al., 1998). This has been corroborated with laboratory studies investigating desiccation resistance in *D. melanogaster* (Folk, 2001). Trehalose is one of the most abundant sugars in insect hemolymph and helps cope with climatic stressors like dehydration (Ring 1998). While clinal variation runs in parallel to latitudinal gradient, an opposing trend is noted for lipids in *D. melanogaster* (Chippindale et al., 1998).

## 3 Genetic and genomic basis of clinal variation

Climate change could trigger changes at the genetic level in populations (Hoffmann and Sgrò 2011). Latitudinal clinal variation for both genetic and genomic levels is known from *Drosophilidae* species of North America, Eastern Australia, and Europe (Adrion et al., 2015; Machado et al., 2021). The clines observed demonstrate parallel variation in alleles and genomic regions for temperate flies and these variations were correlated with seasons. Clines could thus be used to track populations across changing seasons to predict population responses. Clines also exist at the gene level with known variation in markers. For, e.g., latitudinal clines are also observed at the genetic level, at the levels of allozymes, DNA, and chromosomal inversions (Mettler et al., 1977; Knibb 1982) in *Drosophila* species. A case of chromosomal inversion under local selection is that of *D. subobscura* (Balanyá et al., 2006). Clinal variations are also known for the alcohol dehydrogenase (Adh) (also see Table 1) and acetaldehyde dehydrogenase (Aldh) loci (Berry and Kreitman 1993). These enzymes regulating the alcohol detoxification pathway exhibit latitudinal clines in North American *Drosophilidae* populations (Fry et al., 2008).

The trident pigment variation on the thorax regions in *D. melanogaster* has also been explored for its underlying genetic basis (Hoffmann and Sgro 2011; Telonis Scott et al., 2011). Accordingly, the *ebony* locus present in the cosmopolitan inversion *In3R(P)* was found to display clinal variation (Umina et al., 2005). This study confirmed the significance of the *ebony* gene in the clinal variation of trident pigmentation. *D. melanogaster* populations from Northern and Southern Japan were found to differ with respect to the *In*(*3R*)*P* inversions (Inoue and Watanabe 1979), in that southern populations possess higher number of inversions than the northern ones (see Figure 2). Similar trend of *In*(*3R*)*P* inversions was found along latitudinal gradients along the eastern coast of Australia, North America (Knibb 1982) as well as in Asian populations (Singh and Das 1992) of *D. melanogaster*. Chromosomal inversion studies hint that inversions also play a central role in evolutionary processes (through local adaptation) (Kirkpatrick and Barton 2006).

However, clinal patterns can also occur as a result of genetic drift or population history (Hoffmann and Weeks 2007). Genetic drift could cause alleles to be fixed in populations narrowing the cline, but changes within the alleles could broaden the cline (Polechová and Barton 2011). Spatial genetic structure has also been observed along the east coast of Australia and substantial symmetrical gene flow is seen (Gockel et al., 2001). Taylor et al. (2016) in their experiments on *D. pseudoobscura* highlighted the role of female genotype on re-mating. However, the most plausible explanation for the genetic basis of polyandry is the chromosome inversions (Herrera et al., 2014). Interestingly, in many species chromosomal inversions have been found to be impacted by temperature gradients.

## 4 Clinal shifts

Changes in the global climate have led to shifts in the geographical boundaries of the species and not surprisingly in clines as well (Vitousek 1992; Bradshaw and Holzapfel 2008). Studies have tracked the population movements at species borders (Parmesan 1996). Potential impacts of climate change have also been investigated in *Drosophila* (Etges et al., 2006). Several studies also investigated shifts in allele frequency, such as the shifts in the loci of allozymes (Oakeshott et al., 1982). Clinal variations with regards to climate change have been recorded in a variety of organisms (Franks and Hoffmann 2012). Climate changes and phenology (the timing of an organism’s life cycle activities) are interconnected, and change in climate is likely to modify these dynamics (Prendeville et al., 2013). Modification in the environmental gradients leads to shifts in cline position and also phenotypic variation along a species range. This effect is termed as “clinal translocation” by Koch (Koch 1986). One of the best cases of trait shift is the alcohol dehydrogenase (*Adh*) locus in *D. melanogaster* which exhibits a genetic latitudinal cline (Vigue and Johnson 1973). The *Adh* allelic frequency increases with decrease in latitude in the northern as well as southern hemispheres. Variation in the latitudinal cline of the *Adh* has revealed genetic changes correlated to increasing temperatures and drought-related conditions (Umina et al., 2005).

Many organisms are migrating or extending their habitats towards higher latitudes and altitudes in search of cooler temperatures, in response to climate change. Summers are getting warmer, but winters are getting rapidly warm too. The cold of winter is an essential part of many systems in organisms that involve both behavioural and physiological traits. Numerous species of ectotherms from colder climates decrease their energy expenditure which allows them to retain energy stores (Williams et al., 2015). The resource status of an organism in the winter will also determine its performance in the summer that follows. A clinal shift that depicts this phenomenon of migration that shows an extension of an existing cline along Indian latitudes has been observed (Rajpurohit et al. 2008). *D. ananassae* is a tropical warm adapted species but in the last couple of decades has extended its distribution to lowlands of Western Himalayas (Rajpurohit et al., 2008b). This extension of distribution can be explained on the basis of plastic changes which were documented in viability and fecundity correlated with changes in temperature (Rajpurohit et al., 2008a).

## 5 Rapid adaptations

Documented evidence of the impact of rapid climate change on the eco-system demonstrates considerable loss, shifts and alterations in phenology/lifecycles of several species (Pörtner et al., 2022). Evolutionary adaptation can be extremely fast, especially in response to rapidly changing habitats. Rapid adaptation is possible through genetic accommodation or is shaped by natural selection (Shimada et al., 2010). Understanding rapid genomic changes is proving useful to understand how heritability could sustain organisms in the face of climate change. An example is that of *D. subobscura* native to the Old World, but has demonstrated rapid adaptation in wing size, where this trait responds differentially across continents (Huey et al., 2000). *D. subobscura* was introduced to North and South America about two decades ago (Brneie et al., 1981; Beckenbach and Prevosti 1986). Clinal variation with respect to wing size became evident shortly after the first two decades of introduction. It was noted that males from North America displayed less steeper clines for wing size in comparison to their European counterparts; while females had comparable wing measurements across continents. This example demonstrates the rapid pace of trait evolution and adaptation to local selection pressures.

Evolution of cuticular hydrocarbons (CHCs) across short time scales also highlights the physiological adaptation to the changing environment (Rajpurohit et al., 2017a). CHC profiles of *D. melanogaster* across a latitudinal gradient were found to consistently differ both in outdoor mesocosm conditions (fall and spring seasons) and laboratory conditions (Rajpurohit et al., 2017b). Sensitivity to temperature was also confirmed by change in the frequency of alleles across seasons. It is thus interesting to note that response to climate changes could be fast and several gene regulatory networks could be involved. Analyses of single-nucleotide polymorphisms (SNP’s) which could vary at both temporal and spatial scales across latitudinal populations could prove particularly insightful. Recently, (Rudman et al., 2022) populations of *D. melanogaster* were tracked in real time in outdoor conditions (mesocosms), across seasons and adaptation to changing seasons was evident through changes in allele frequency. If allele frequencies could change across seasons, long term seasonal changes across years could also be investigated for similar changes (Figure 1). Indeed Behrman et al. (2015) showed that seasonally varying SNP’s could be maintained by balancing selection. Data collected from *D. melanogaster* populations in temperate orchards (North America) revealed that SNP oscillation across seasons could be adaptive in response to extreme frost as well warming conditions. Whole genome comparisons demonstrate a 2.5% increased frequency in SNPs of northern associated alleles indicating the presence of latitudinal cline. Rapid adaptation is thus evident in the phase of climate change with rising temperatures.

Signaling pathways involved in developmental wiring are seen as likely candidates to understand the molecular basis of rapid adaptation. Selection acting on genetic variation differs across time scales. Some genetic variations might be directionally selected, while some would be affected by fluctuating environments over shorter time scales (Rudman et al., 2022). Nevertheless sampling at regular time-points could yield insights into the neutral/non-neutral basis of selection (Lange et al., 2022).

## 6 Gaps and future prospects

Ectotherms (insects, amphibians, reptiles) are particularly sensitive to thermal fluctuations and rapid climate change could often stretch the thermal limits outside the optimal range (Kingsolver et al., 2013). Unlike mammals or large organisms, the comparatively small size of insects is not a constraint in migration. Hence more studies investigating clines related to life cycle stages (especially juvenile) could help understand adaptive responses, e.g., faster growth at larval stage, diapause induction and even energy balance (metabolites). Low temperatures induce diapause in temperate regions. Global warming could either break this diapause earlier or may not induce diapause at all. Adults eclosing in mismatched environments could face survival challenges since seasonal changes could still be governed by photoperiod. It would be interesting to note if insects in temperate versus tropics have supernumerary (extra) generations as a consequence of climate change. Clinal exploration in ectotherms could thus be useful in tracing the rapid impact of climate on life history traits. The “Climate variability hypothesis” (CVH) proposed by Stevens (1989) posits that temperate species will be more capable of expanding their geographical range of distribution than tropical species (Sheldon and Tewksbury 2014). This indicates a positive correlation between the range of thermal tolerance and increasing latitude, which needs to be further explored (Addo-Bediako et al., 2000). Climatic fluctuation plays a significant role in determining the ecological range of species (Currie et al., 2004). Between 1970 and 2000 more than half of the species have responded to warming globally. This contribution can also be indirect *via* alterations in species’ interactions (Thomas 2010). To see how climate change influences clines in closely interacting species, an experiment was conducted by Davis et al. (1998) wherein three *Drosophila* species were exposed to simulated global warming conditions. At low temperatures, dispersal enhanced fitness in *D. subobscura* compared to *D. melanogaster* and *D. simulans*. However, at high temperatures, fitness of all three species was compromised. It is thus becoming clear that climate change would induce habitat changes, but interaction with other biotic factors could also influence organismal fitness. Glancing through existing literature, we present few perspectives on clinal variation which might yield further insights into species response to climate change:

### 6.1 Revisiting older clines, find comparative traits

Clines: both phenotypic and genomic are largely known from the temperate zones for Drosophilids (Adrion et al., 2015). Little is known about the genetic bases for clines in Africa and Asia. It would be insightful to investigate the genetic and genomic basis of clinal variation observed in Africa and Asia (Rajpurohit et al., 2022) and compare these with temperate counterparts. Perhaps the complex network of biotic interactions available in tropical and sub-tropical Asia and Africa could mean populations have options for relocation, alternate host/food sources and thus lowered vulnerability to extinction. It is then interesting to note if parallel differentiation is common across latitudinally similar clines (even with tropics). Considering the global diversity in the tropics, one would expect variation in both the number of traits exhibiting clines and the spread (level of gradient) between/among these clines; to differ from the temperate species. Thus, clinal variation for stress resistance traits are more common across the Indian subcontinent (tropical), while diapause clines were more frequently associated with temperate climate regimes (see Table 1 and Supplementary Table S1). This could be due multiple ecological factors selecting a particular phenotype. Local selection pressures other than temperature, e.g., hypoxia, moisture content, predation, and biological interactions could also likely select traits optimizing fitness for a given environment.

### 6.2 Investigating wide spread clines

Clinal variation in several cases is known from extreme phenotypes. However, the magnitude of variation within a cline remains largely unknown. In the face of climate change, populations from widespread clines could be expected to have higher chances of survival even if populations from a particular zone get wiped out or face extinction. It could be relevant to explore geographic zones spanning variation in latitudes and altitudes with known clines. For example, the Indian subcontinent (Rajpurohit et al., 2017b) offers a series of graded environments from cold to humid habitats and elevations ranging from coasts to 3,000 m above sea level which hitherto remain under-explored.

Global warming predicts that species will migrate poleward or towards higher altitudes as temperatures rise. To what extent will this modify/relocate/shift clines? For example, will pigmentation/body size for populations from lower latitudes/altitudes increase further when they migrate towards higher latitudes/altitudes? The clear answer to this question is we do not know. If there is no heritable variation for a particular trait, organisms would not be able to adapt even if they relocate to different geographical locations. It is therefore important to study the genetic basis of clinal variation broadly across species and geographic scales. It would also be interesting to connect clinal traits with the critical thermal tolerances of organisms since tropical species could have narrower thermal tolerances than temperate species (Kellermann et al., 2012). If this is the case do tropical populations migrate poleward or will latitudinal clines shrink? How high would temperate populations migrate in the face of climate change? These remain open ended questions and studies linking existing clines, migration events could shed more light.

Rapid adaptations present a new area to explore the evolution of clines. Thus, diapause induction or increased tolerance to cold was not observed for the ancestral population of *D. melanogaster* from Africa. However, recently migrated *D. melanogaster* populations in North America (Schmidt and Conde, 2006) and Australia (Collinge et al., 2006) exhibit positive clines for diapause/cold tolerance than African ancestors. It is therefore likely that *D. melanogaster* dispersing flies from Europe to North America passed these differentiated loci (in ca. 200 years) to North America and Australia (also see Figure 2).

### 6.3 Disconnect between morphological and molecular analysis and long term tracking

The plasticity component for every trait needs to be well characterized, because these could likely have cross talks with climatic variables. A plastic trait could enable fitness across diverse environments only through environmental effects (only E and not GXE). However, unless the trait is heritable, the plastic component will not be adaptive in successive generations. Long-term studies using biomarkers, merging both molecular and physiological aspects, could be used to track loss of biodiversity and spread of populations in the face of climate change.

## 7 Conclusion

Species unable to adapt to climate change risk extinction. While climate change has been known through millenia, the pace has been faster in recent years. Rapid increase in temperature, a key factor of climate change presents a challenge for organismal survival. Organisms from lower latitudes and altitudes are more likely to go extinct than those from higher altitudes and latitudes. An understanding of how organisms across latitudes/altitudes are responding to climate change could thus be crucial in implementing preventive measures, e.g., conservation, reducing biodiversity loss. We therefore propose clinal variation studies to complement measures of tackling climate change. Given their speciose nature and wide distribution, insects seem promising systems to study climate mediated changes. Existing knowledge from morphological and life history traits provides some intriguing clues. However, broader-scale comparisons of genetic and plastic traits are still less known. We therefore focus on the amenable (high-throughput platform available) fruit fly model system, which could serve to explore adaptations to climate change through key traits linked with survival and fitness. Molecular studies reveal allelic changes in response to temperature fluctuation spanning even shorter time-periods in fruit flies. We discuss clinal variation with respect to a wide variety of traits which could affect species fitness and also response to climate change (Figure 1). We highlight potential shortcomings in clinal studies and suggest how these could be addressed to understand species redistribution, clinal shift patterns. Overall our review emphasizes the necessity to understand climatic change through an alternative lens of clinal variation, less explored till now.
