# Supplementary material for: Clinal variation as a tool to understand climate change
Source: Front Physiol. 2022 Oct 11;13:880728. doi: 10.3389/fphys.2022.880728 (PMC9593049; doi:10.3389/fphys.2022.880728)
Supplement: Supplementary file 1 [file Table1.docx]

**Supplementary Table 1**: The studies cited in Table 1 in the main text have been referenced in detail here. Species, geographical scales (altitude/latitude), traits with respective publications have been tabulated below. The genus Drosophila has been abbreviated *D*. Co-ordinates of the range of latitudes and altitudes from where populations were obtained have been mentioned. Numbers in parentheses refer to the number of populations collected from either a single locality or from multiple locations.

| **Continents** | **Species** | **Geographic scale** | | **Traits** | **References** |
| --- | --- | --- | --- | --- | --- |
|  | | **Latitudinal** | **Altitude (a.s.l)** |  | |
| **Australia** | *D.melanogaster, D. simulans* | Melbourne ([37°48S 144°57′47″E](https://geohack.toolforge.org/geohack.php?pagename=Melbourne&params=37_48_49_S_144_57_47_E_region:AU-VIC_type:city(5,200,000))) (1) |  | Desiccation tolerance | (McKenzie & Parsons, 1974) |
|  | *D.melanogaster* |  | 300m to 2100m  27.6728° S, 148.3717° E | Body Size | (James *et al.*, 1995) |
|  | *D.melanogaster* | 16" 53 - 42" 53' ^o^S  (20) |  | Larval Development (Time) | (James & Partridge, 1995) |
|  | *D.melanogaster* | 17°- 43^o^ S (20) |  | Wing Thorax ratio | (Azevedo *et al.*, 1998) |
|  | *D. serrata* | 36^o^S – 16^o^S  (2) |  | Chill coma, body size | (Hallas *et al.*, 2002) |
|  | *D. melanogaster* | Coast East  35” 49’N |  | Body Size | (James *et al.*, 1995),  (Imasheva *et al.*, 1994) |
|  | *D.melanogaster* | Australia (20) |  | Ovariole Number | (Azevedo *et al.*, 1996),  (Schmidt *et al.*, 2005) |
|  | *D.melanogaster* | 43^o^6’ S- 15^o^28’ S |  | Cold resistance | (Hoffmann & Alisha Anderson and Rebecca Halla, 2002) |
|  | *D.melanogaster* | 25.27° S, 133.77° E.  42.04° S, 146.81° E |  | Wolbachia Infection Frequency | (Kriesner *et al.*, 2016) |
|  | *D.melanogaster* | Tropical: 16.907^o^S, 145.709^o^E, 18.267 ^o^S, 146.017^o^E.  Temperate: 42.769 ^o^S, 147.576^o^E (1) |  | Day time sleep (Siesta) | (Yang & Edery, 2018) |
| **Asia** | *D.melanogaster, D. simulans* | Japan  (35.6635° N, 138.7317° E) (7) |  | Ovariole Number | (Metropolitan, 1986) |
|  | *D.melanogaster, D. ananassae, Zaprionus indianus* | India  9.2° - 28.9°  (9) |  | Desiccation/ Starvation Tolerance | (Karan *et al.*, 1998a) |
|  | *D.melanogaster, D. kikkawai* | India, Sri Lanka  6.8^o^N – 31.3^o^N |  | Wing length | (Karan *et al.*, 1998b) |
|  | *D.melanogaster* | India  10 – 32^o^74’N | 600– 2050m  (30.46′ -32.3′°N) (5) | Trident pigmentation | (Parkash & Munjal, 1999b) |
|  | *D.melanogaster, D. kikkawai,*  *D.* ananassae*,*  *D. bipectinata,*  *D. repleta* | 6.6 ^o^N – 31.3 ^o^N (5) |  | Body weight | (Karan *et al.*, 1998b) |
|  | *D.melanogaster, D. ananassae,*  *Z. indianus,*  *D. repleta* | 10.0 ^o^N to 32.7 ^o^N  (4) |  | Desiccation resistance | (Parkash & Munjal, 1999a) |
|  | *D.melanogaster* | 17.3′ – 32.3′°N (9) |  | Desiccation tolerance, Starvation resistance | (Karan *et al.*, 1998a) |
|  | *D.melanogaster* |  | 10– 2202m  (11.15′ – 31.06′°N)  (10) | Ovariole number | (Rajpurohit *et al.*, 2008) |
|  | *D. takahashi,*  *D. nepalenesis* |  | 219 – 2202 m | Desiccation tolerance, Starvation resistance | (Parkash *et al.*, 2005) |
|  | *D. nasuta,*  *D. immigrans* | 11.0 – 30.51°N (7) |  | Chill-coma recovery,  Heat-knockdown resistance | (Parkash & Ranga, 2013) |
|  | *D. immigrans* | 30.51′°N – 32.36′°N | 600 – 2226m (8) | Abdominal pigmentation, Desiccation resistance | (Parkash *et al.*, 2008) |
|  | *D. kikkawai* | 12.6′–32.7′°N (7) |  | Desiccation tolerance, Starvation resistance | (Karan *et al.*, 1998a) |
|  | *D. kikkawai* | 8.29′°N – 32.7′°N (8) |  | Body weight,  Wing length,  Thorax length,  Abdominal bristles, Ovariole number | (Parkash & Munjal, 1999a) |
|  | *D. kikkawai* | 6.8′°N – 31.3′°N (9) |  | Thorax length,  Wing length,  Sterno-pleural bristles, Abdominal bristles,  Body weight,  Abdominal pigmentation | (Karan, 1998) |
|  | *D. melanogaster* | 8.29 – 32.40°N (8) |  | Total body lipids,  Trehalose | (Parkash *et al.*, 2012) |
|  | *D. melanogaster, D. simulans* | Southern Turkey  50 – 1500 m  (37.03 – 38.25°N) (4) |  | Body size,  Chill coma recovery | (Yilmaz& Özsoy, 2022) |
|  | *D.melanogaster* | 30.50 °N – 8.05 °N |  | Copulation duration | (Parkash *et al.*, 2011) |
| **South America** | *D.melanogaster* | 2^o^ 13^o^S, 41°30^o^S  (10 P) |  | Body Size | (van ’T Land *et al.*, 1999) |
|  | *D. subobscura* | 29.92 S - 56.2 N  (2p) |  | Wing Size | (Calboli *et al.*, 2003) |
|  | *D.melanogaster* | 2^o^00’S 77 ^o^30’W  (1) | **_** | Egg size | (Azevedo *et al.*, 1996) |
|  | *D.subobscura*  *(Female)* | -45^o^35- 56^o^9’N. (13) |  | Wing Size | (Gilchrist *et al.*, 2004) |
|  | *D.buzzatti* |  | North western Argentina  26^o^36’S, 65 ^o^ 52’W; 2300m,  27 ^o^ 48’S, 64 ^o^ 18’W; 202 m | Thermal tolerance | (Sørensen *et al.*, 2005) |
| **North America** | *D.subobscura*  *(Females)* | 35.5^o^N - 50.7^o^N (11) |  | Wing length | (Huey, 2000) |
|  | *D. subobscura* | 35.5^o^N -50.7^o^N (1) |  | Cell size | (Calboli *et al.*, 2003) |
|  | *D.melanogaster* | Walpole (43.9522° N, 69.5598° W) (1) |  | Diapause | (Schmidt & Conde, 2006) |
|  | *D.melanogaster* | 35.7796° N, 78.6382° W  North Caroline (Raleigh) USA. (1) |  | Aldh-Phe gene | (Fry, 2001) |
|  | *D. subobscura* | 37^o^0’21” N, 38^o^31’1” N, 40^o^46’41” N (1) |  | Thermoregulatory Behaviour | (Huey, 2009) |
|  | *D. melanogaster* | 25°32′N, 80°29′W, 39°53′N, 75°24′W. 44°1′N, 69°56′  (1) |  | In (3R) Payne | (Fabian *et al.*, 2012) |
|  | *D. dunni* | 18.22° N, 66.59° W (1) |  | Abdominal Pigmentation | (Hollocher *et al.*, 2046) |
| **Europe** | *D.subobscura*  *(Females)* | 36.5^o^N - 56.1^o^N (10) |  | Wing length | (Huey, 2000) |
|  | *D.buzzatti* |  | 28.29° N, 16.63° W (9) | Starvation resistance | (Sarup & Loeschcke, 2010) |
|  | *D.melanogaster* | 36.97^o^N – 62.01 ^o^N (6) |  | Time less allele (ls-Tim) | (Pegoraro *et al.*, 2017) |
|  | *D. melanogaster* | -33.72^o^N -59.85^o^N | 7 m - 3050 m | Ovariole number, Wing size and Cell number | (Klepsatel et al., 2014) |
| **Africa** | *D.melanogaster* |  | 295 m – 2506 m (1) | Wing size | (Pitchers *et al.*, 2013) |
|  | *D. melanogaster* |  | 78-2506 m (20) | Mid-day siesta | (Cao & Edery, 2017) |
|  | *D. melanogaster* | -33.72^o^N -59.85^o^N | 7 m - 3050 m | Ovariole number, Wing size and Cell number | (Klepsatel *et al.*, 2014) |
|  | *D. melanogaster* | 7.37° N, 12.35° E (Cameroon)  3.22° S, 40.12° E  (Kenya, Malindi)  1.37° N, 32.29° E  (Uganda) |  | Abdominal pigmentation | (Pool and Aquadro 2007) |

**References:**

Azevedo, R.B.R., French, V. & Partridge, L. 1996. Thermal evolution of egg size in Drosophila melanogaster. *Evolution (N Y)***50**: 2338–2345.

Azevedo, R.B.R., James, A.C., McCabe, J. & Partridge, L. 1998. Latitudinal variation of wing: thorax size ratio and wing-aspect ratio in Drosophila melanogaster. *Evolution (N Y)***52**: 1353–1362.

Calboli, F.C.F., Gilchrist, G.W. & Partridge, L. 2003. Different cell size and cell number contribution in two newly established and one ancient body size cline of Drosophila subobscura. *Evolution (NY)***57**: 566–573.

Cao, W. & Edery, I. 2017. Mid-day siesta in natural populations of D. melanogaster from Africa exhibits an altitudinal cline and is regulated by splicing of a thermo sensitive intron in the period clock gene. *BMC EvolBiol***17**: 1–17. BioMed Central.

Fabian, D.K., Kapun, M., Nolte, V., Kofler, R., Schmidt, P.S., Schlötterer, C., *et al.* 2012. Genome-wide patterns of latitudinal differentiation among populations of Drosophila melanogaster from North America. *MolEcol***21**: 4748–4769.

Fry, J.D. 2001. Direct and correlated responses to selection for larval ethanol tolerance in Drosophila melanogaster. *J EvolBiol***14**: 296–309.

Gilchrist, G.W., Huey, R.B., Balanyà, J., Pascual, M. & Serra, L. 2004. A time series of evolution in action: A latitudinal cline in wing size in South American Drosophila subobscura. *Evolution (N Y)***58**: 768–780.

Hallas, R., Schiffer, M. & Hoffmann, A.A. 2002. Clinal variation in Drosophila serrata for stress resistance and body size. *Genet Res***79**: 141–148.

Hoffmann, A.A. & Alisha Anderson and Rebecca Halla. 2002. <Hoffmann et al 2002 - Opposing clines for high and low temperature resistance in Drosophila melanogaster.pdf>. 614–618.

Hollocher, H., Hatcher, J.L. &Dyreson, E.G. 2046. *EVOLUTION OF ABDOMINAL PIGMENTATION DIFFERENCES ACROSS SPECIES IN THE DROSOPHILA DUNNI SUBGROUP*.

Huey, R.B. 2000. Rapid evolution of a geographic cline in size in an introduced fly. *Science (1979)***287**: 308–309.

Huey, R.B. and M.P. 2009. R eports R eports. **90**: 1715–1720.

Imasheva, A.G., Bubli, O.A. &Lazebny, O.E. 1994. Variation in wing length in eurasian natural populations of drosophila melanogaster. *Heredity (Edinb)***72**: 508–514.

James, A.C., Azevedo, R.B.R. & Partridge, L. 1995. Cellular basis and developmental timing in a size cline of Drosophila melanogaster. *Genetics***140**: 659–666.

James, A.C. & Partridge, L. 1995. Thermal evolution of rate of larval development in Drosophila melanogaster in laboratory and field populations. *J EvolBiol***8**: 315–330.

Karan, D. 1998. Desiccation tolerance and starvation resistance exhibit opposite latitudinal clines in Indian geographical populations of Drosophila kikkawai. *EcolEntomol***23**: 391–396.

Karan, D., Dahiya, N., Munjal, A.K., Gibert, P., Moreteau, B., Parkash, R., *et al.* 1998a. Desiccation and starvation tolerance of adult Drosophila: Opposite latitudinal clines in natural populations of three different species. *Evolution (N Y)***52**: 825–831.

Karan, D., Munjal, A.K., Gibert, P., Moreteau, B., Parkash, R. & David, J.R. 1998b. Latitudinal clines for morphometrical traits in Drosophila kikkawai: A study of natural populations from the Indian subcontinent. *Genet Res***71**: 31–38.

Klepsatel, P., Gáliková, M., Huber, C.D. &Flatt, T. 2014. Similarities and differences in altitudinal versus latitudinal variation for morphological traits in drosophila melanogaster. *Evolution (N Y)***68**: 1385–1398. Society for the Study of Evolution.

Kriesner, P., Conner, W.R., Weeks, A.R., Turelli, M. & Hoffmann, A.A. 2016. Persistence of a Wolbachia infection frequency cline in Drosophila melanogaster and the possible role of reproductive dormancy. *Evolution (N Y)***70**: 979–997.

McKenzie, A. & Parsons, P.A. 1974. The genetic architecture of resistance to desiccation in populations of drosophila melanogaster and d. simulans. *Aust J BiolSci***27**: 441–456.

Metropolitan, T. 1986. Jpn. J. Genet. (1986) 61, pp. 469-480. 469–480.

Parkash, R., Aggarwal, D.D. &Kalra, B. 2012. Coadapted changes in energy metabolites and body color phenotypes for resistance to starvation and desiccation in latitudinal populations of D. melanogaster. *EvolEcol***26**: 149–169.

Parkash, R.& Munjal, A.K. 1999a. Climatic selection of starvation and desiccation resistance in populations of some tropical drosophilids. *Journal of Zoological Systematics and Evolutionary Research***37**: 195–202.

Parkash, R. & Munjal, A.K. 1999b. Phenotypic variability of thoracic pigmentation in indian populations of Drosophila melanogaster. *Journal of Zoological Systematics and Evolutionary Research***37**: 133–140.

Parkash, R., Ramniwas, S., Rajpurohit, S. & Sharma, V. 2008. Variations in body melanization impact desiccation resistance in Drosophila immigrans from Western Himalayas. *J Zool***276**: 219–227.

Parkash, R. & Ranga, P. 2013. Divergence for tolerance to thermal-stress related traits in two Drosophila species of immigrans group. *J ThermBiol***38**: 396–406.

Parkash, R., Sharma, V., Chahal, J., Lambhod, C. &Kajla, B. 2011. Impact of body melanization on mating success in Drosophila melanogaster. *EntomolExpAppl***139**: 47–59.

Parkash, R., Tyagi, P.K., Sharma, I. & Rajpurohit, S. 2005. Adaptations to environmental stress in altitudinal populations of two Drosophila species. *PhysiolEntomol***30**: 353–361.

Pegoraro, M., Zonato, V., Tyler, E.R., Fedele, G., Kyriacou, C.P. &Tauber, E. 2017. Geographical analysis of diapause inducibility in European Drosophila melanogaster populations. *J Insect Physiol***98**: 238–244. Elsevier Ltd.

Pitchers, W., Pool, J.E. &Dworkin, I. 2013. Altitudinal clinal variation in wing size and shape in african drosophila melanogaster: One cline or many? *Evolution (N Y)***67**: 438–452.

Pool, J. E. and C. F. Aquadro (2007). "The genetic basis of adaptive pigmentation variation in Drosophila melanogaster." Mol Ecol **16**(14): 2844-2851.

Rajpurohit, S., Parkash, R., Ramniwas, S. & Singh, S. 2008. Variations in body melanisation, ovariole number and fecundity in highland and lowland populations of Drosophila melanogaster from the Indian subcontinent. *Insect Sci***15**: 553–561.

Sarup, P. & Loeschcke, V. 2010. Developmental acclimation affects clinal variation in stress resistance traits in Drosophila buzzatii. *J EvolBiol***23**: 957–965.

Schmidt, P.S. & Conde, D.R. 2006. Environmental Heterogeneity and the Maintenance of Genetic Variation for Reproductive Diapause in Drosophila Melanogaster. *Evolution (N Y)***60**: 1602.

Schmidt, P.S., Matzkin, L., Ippolito, M. &Eanes, W.F. 2005. Geographic Variation in Diapause Incidence , Life-History Traits , and Climatic Adaptation in Drosophila melanogaster Author ( s ): Paul S . Schmidt , Luciano Matzkin, Michael Ippolito and Walter F . Eanes Published by : Society for the Study of Evolutio. *Evolution (N Y)***59**: 1721–1732.

Sørensen, J.G., Norry, F.M., Scannapieco, A.C. & Loeschcke, V. 2005. Altitudinal variation for stress resistance traits and thermal adaptation in adult Drosophila buzzatii from the New World. *J EvolBiol***18**: 829–837.

van ’T Land, J., van Putten, P., Zwaan, B., Kamping, A. & van Delden, W. 1999. Latitudinal variation in wild populations of Drosophila melanogaster: Heritabilities and reaction norms. *J EvolBiol***12**: 222–232.

Yang, Y. & Edery, I. 2018. Parallel clinal variation in the mid-day siesta of Drosophila melanogaster implicates continent-specific targets of natural selection. *PLoS Genet***14**: 1–25.

YILMAZ, M. & ÖZSOY, E.D. 2022. Altitudinal variation in body size and resistance to stress in Drosophila melanogaster and D. simulans (Diptera: Drosophilidae) in southern Turkey. *Eur J Entomol***119**: 140–147.
